# Atypical teratoid rhabdoid tumor: improved long-term survival with an intensive multimodal therapy and delayed radiotherapy. The Medical University of Vienna Experience 1992–2012

**DOI:** 10.1002/cam4.161

**Published:** 2013-12-11

**Authors:** Irene Slavc, Monika Chocholous, Ulrike Leiss, Christine Haberler, Andreas Peyrl, Amedeo A Azizi, Karin Dieckmann, Adelheid Woehrer, Christina Peters, Georg Widhalm, Christian Dorfer, Thomas Czech

**Affiliations:** 1 Department of Pediatrics, Medical University of ViennaVienna, Austria; 2 Institute of Neurology, Medical University of ViennaVienna, Austria; 3 Department of Radiotherapy, Medical University of ViennaVienna, Austria; 4 SCT Unit, St. Anna KinderspitalVienna, Austria; 5 Department of Neurosurgery, Medical University of ViennaVienna, Austria

**Keywords:** ATRT, delayed local radiotherapy, high-dose chemotherapy, improved survival, multimodal therapy

## Abstract

Atypical teratoid rhabdoid tumors (ATRTs) are recently defined highly aggressive embryonal central nervous system tumors with a poor prognosis and no definitive guidelines for treatment. We report on the importance of an initial correct diagnosis and disease-specific therapy on outcome in 22 consecutive patients and propose a new treatment strategy. From 1992 to 2012, nine patients initially diagnosed correctly as ATRT (cohort A, median age 24 months) were treated according to an intensive multimodal regimen (MUV-ATRT) consisting of three 9-week courses of a dose-dense regimen including doxorubicin, cyclophosphamide, vincristine, ifosfamide, cisplatin, etoposide, and methotrexate augmented with intrathecal therapy, followed by high-dose chemotherapy (HDCT) and completed with local radiotherapy. Thirteen patients were treated differently (cohort B, median age 30 months) most of whom according to protocols in use for their respective diagnoses. As of July 2013, 5-year overall survival (OS) and event-free survival (EFS) for all 22 consecutive patients was 56.3 ± 11.3% and 52.9 ± 11.0%, respectively. For MUV-ATRT regimen-treated patients (cohort A) 5-year OS was 100% and EFS was 88.9 ± 10.5%. For patients treated differently (cohort B) 5-year OS and EFS were 28.8 ± 13.1%. All nine MUV-ATRT regimen-treated patients are alive for a median of 76 months (range: 16–197), eight in first complete remission. Our results compare favorably to previously published data. The drug combination and sequence used in the proposed MUV-ATRT regimen appear to be efficacious in preventing early relapses also in young children with M1–M3 stage disease allowing postponement of radiotherapy until after HDCT.

## Introduction

Atypical teratoid rhabdoid tumors (ATRTs) are rare, highly aggressive embryonal central nervous system (CNS) tumors primarily encountered in children with a peak incidence in infants less than 3 years of age 1. Rhabdoid tumors were originally described as an aggressive variant of Wilms tumors with rhabdomyosarcomatous features and subsequently observed also in soft tissues and the CNS 2. ATRTs were defined as an entity in 1996 3 and added to the World Health Organization (WHO) brain tumor classification in 2000 4. Histopathologically, ATRTs are characterized by rhabdoid tumor cells and varying amounts of small undifferentiated primitive neuroectodermal tumor (PNET)-like, mesenchymally, and/or epithelially differentiated tumor cells. The vast majority of ATRTs are characterized by alterations of the *SMARCB1* (*hSNF5*/*INI1*) gene at chromosomal locus 22q11.23, resulting in loss of nuclear protein expression. Consistent with the role of a tumor suppressor gene, biallelic inactivation of *SMARCB1* is present in rhabdoid tumors, and ATRTs may occur sporadically or in the setting of a rhabdoid predisposition syndrome 5,6.

Before the introduction of an antibody directed against the SMARCB1/INI1 protein into routine diagnostics in 2004 7, ATRTs were frequently misdiagnosed if characteristic rhabdoid tumor cells were missing in the biopsy specimen 8. The most frequent misdiagnosis of ATRT was medulloblastoma or CNS PNET, particularly when a primitive neuroepithelial component was prominent 1,9.

Historically, the prognosis of patients with ATRT was poor with a median survival of approximately 1 year 10–16. Due to the rarity of the disease and the lack of large formal prospective trials, the patients were usually treated in a heterogeneous manner, and no definitive guidelines for optimal treatment have been established. More recently, long-term survivors including patients with recurrent and disseminated disease have been reported, and disease-specific protocols and registries combining maximal surgical resection, intensive chemotherapy with or without stem cell support, and radiotherapy have been opened and show encouraging, albeit preliminary results 17–19.

We report on the importance of an initial correct diagnosis and consequently disease-specific therapy on outcome in 22 consecutive patients with ATRT, treated at the Medical University of Vienna between 1992 and 2012, and propose a new treatment strategy.

## Patients and Methods

A retrospective analysis applying the SMARCB1/INI1 antibody to all highly malignant pediatric brain tumors treated at the Medical University of Vienna (MUV) since 1992 disclosed 22 patients with ATRT. The clinical characteristics of the patients are shown in Table 1.

**Table 1:** Patient characteristics, type of therapy, and outcome.

Case		Age at DX (months/years), gender	Year of DX	Primary tumor location	Chang stage	Extent of resection	Original DX	SMARC B1	Chemotherapy	Intrathecal chemotherapy	HDCT	Radiotherapy	Response to CT	Time to relapse/progress (months)	Duration of survival (months)	Disease status
*Cohort A*																
1	6 years	F	1997	Frontal	M0	STR	ATRT	Neg.	MUV-ATRT	–	+;	Focal	CR	–	197	NED
2	24 months	M	2002	Post. fossa	M0	GTR	ATRT	Neg.	MUV-ATRT	Double	+;	Focal	n.e.	–	132	NED
3	12 months	M	2002	Post. fossa	M0	GTR	ATRT	Neg.	MUV-ATRT	Double	+;	Focal	n.e.	(114)^1^	127	NED
4	9 months	M	2005	Post. fossa	M3	PR	ATRT	Neg.	MUV-ATRT	Triple	+;	Focal	CR	–	98	NED
5	23 months	M	2007	Post. fossa	M1	STR	ATRT	Neg.	MUV-ATRT	Triple	+;	Focal	PR	–	76	NED
6	20 months	M	2009	Post. fossa	M1	GTR	ATRT	Neg.	MUV-ATRT	Triple	+;	Focal	CR	–	53	NED
7	56 months	M	2009	3rd ventr.	M1	GTR	ATRT	Neg.	MUV-ATRT	Double	+;	Focal	CR	16	48	NED
8	17 years	F	2009	Temporal	M0	STR	ATRT	Neg.	MUV-ATRT	Double	+;	Focal	CR	–	44	NED
9	13 years	M	2012	Thalamus	M0	GTR	ATRT	Neg.	MUV-ATRT	Double	+;	Focal	n.e.	–	16	NED
*Cohort B*																
1	7 months	F	1992	Post. fossa	M0	GTR	EPB	Neg.	HIT-SKK-92	MTX	−	No	PD	3	8	DOD
2	2 months	M	1993	Post. fossa	M0	GTR	MB	Neg.	–	–	−	No	n.e.	–	0	DOD
3	20 months	M	1993	Post. fossa	M0	STR	MB	Neg.	HIT-SKK-92	MTX	−	No	PR	6	8	DOD
4	9 years	F	1994	Basal ganglia	n.a.	PR	PNET	Neg.	HIT-91	Mafo	−	CSI +; GKN	CR	(174)^1^	200	DOC
5	2 months	M	1998	Post. fossa	M3	STR	MB	Neg.	HIT-SKK-92	MTX	−	No	PD	3	5	DOD
6	4 months	M	1998	Post. fossa	M3	GTR	EPB	Neg.	HIT-SKK-92	MTX	−	No	PD	3	7	DOD
7	39 months	M	1999	Spinal	M0	Biopsy	ATRT	Neg.	4 × PEI	–	+;	Focal	CR	18	24	DOD
8	30 months	F	1999	Spinal	M1	Biopsy	EWS	Neg.	4 × PEI	–	−	Focal	PR	4	6	DOD
9	14 months	F	1999	Pineal/midbrain	M0	GTR	PNET	Neg.	HIT-SKK-92	MTX	+;	No	n.e.	46	56	DOD
10	35 months	M	2005	Thalamus	n.a.	PR	GBM	Neg.	8 × PEI	–	−	Focal	CR	–	100	NED
11	14 years	F	2006	Frontal	n.a.	STR	ATRT	Neg.	n.a.^2^	–	−	CSI	n.e.	–	7	DOC
12	22 years	M	2007	Frontal	M0	GTR	PNET	Neg.	HIT-2000	–	−	CSI	n.e.	–	74	NED
13	11 years	F	2010	Fronto-par.	M0	STR	ATRT	Neg.	MUV-ATRT^3^	Double	−	CSI	SD	–	34	SD

DX, diagnosis; HDCT, high-dosage chemotherapy; CT, chemotherapy; F, female; M, male; n.e., not evaluable; post., posterior; ventr., ventricle; n.a., not available; STR, subtotal resection; GTR, gross total resection; PR, partial resection/partial remission; MUV-ATRT, Medical University of Vienna ATRT-protocol; MTX, methotrexate; Mafo, mafosfamide; CSI, craniospinal irradiation; GKN, gamma knife; CR, complete remission; PD, progressive disease; NED, no evidence of disease; DOD, dead of disease; DOC, death of other cause; AWD, alive with disease; EPB, ependymoblastoma; MB, medulloblastoma; ATRT, atypical teratoid rhabdoid tumor; PNET, CNS primitive neuroectodermal tumor; EWS, Ewing sarcoma; GBM, glioblastoma multiforme.

1 Secondary malignancy.

2 Patient moved to another country.

3 Intention-to-treat (changed to CSI because of delay in CT following septicemia).

Nine of the patients originally diagnosed correctly (cohort A) were treated uniformly according to the same prospective strategy used in a first index patient diagnosed in 1997 who had become a long-term survivor. Thirteen patients were treated differently (cohort B), most of whom according to protocols for their respective diagnoses.

### Age at diagnosis and gender

Median age at diagnosis was 24 months (range: 9 months–17 years) in cohort A and 30 months (range: 2 months–22 years) in cohort B. The male:female ratio was 1.8:1 for all patients.

Tumor location, staging, and degree of surgical resection are shown in Table 1. Fifteen patients received an Ommaya reservoir, in one of these patients, who was shunt-dependent, Ommaya reservoir placement was combined with exchanging the shunt to a valve with an integrated on–off device 20.

### Histopathology

For histopathology review, conventional histological stainings and the immunohistochemical analysis of SMARCB1 protein expression were performed as previously described 8. According to the current WHO classification of tumors of the CNS 21 ATRT was diagnosed when rhabdoid tumor cells were present and/or divergent differentiation along epithelial, mesenchymal, neuronal, or glial lines were found, and when complete loss of SMARCB1 protein expression was observed in tumor cell nuclei, but expression was retained in preexisting cells (e.g., endothelial cells).

### Chemotherapy

**Figure 1:**
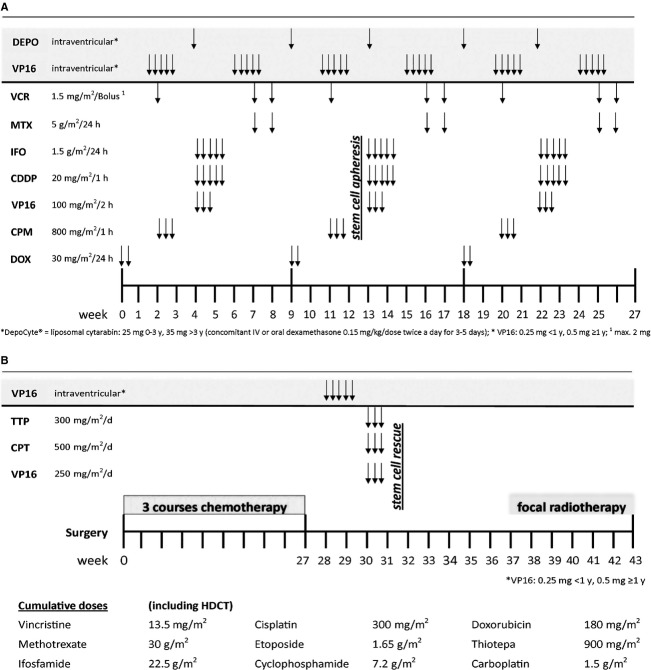
MUV-ATRT regimen for newly diagnosed ATRT patients. (A) Week 0–27. (B) Week 28–43 and cumulative doses. MUV-ATRT, Medical University of Vienna ATRT-protocol; ATRT, atypical teratoid rhabdoid tumor.

Following surgery all but one patient, who died shortly after surgery, received further antitumor therapy. For patients with an initial correct diagnosis, a multimodal treatment strategy which was successful in a first index patient became the standard of treatment for all except two consecutive cases since 2002. The proposed treatment strategy (MUV-ATRT regimen) consisted of three 9-week courses of a dose-dense regimen including doxorubicin, cyclophosphamide, vincristine, ifosfamide, cisplatin, etoposide, and high-dose methotrexate (HD-MTX), augmented with intrathecal therapy and followed by high-dose chemotherapy (HDCT) with autologous hematopoietic stem cell reinfusion according to a modified Finlay protocol (carboplatin 500 mg/m^2^, etoposide 250 mg/m^2^, and thiotepa 300 mg/m^2^ given simultaneously from Day 6 to Day 4) 22. Treatment was completed with local radiotherapy starting 6 weeks after HDCT (Fig. 1A and B). The two exceptions were a 14-year-old patient (case 11, cohort B) who moved to another country and an 11-year-old patient (case 13, cohort B) who was operated in another hospital and was switched to craniospinal irradiation following major postoperative infectious complications and poor bone marrow tolerance.

Most patients in cohort B were treated according to the HIT brain tumor protocols proposed by the German Society for Paediatric Oncology and Haematology (GPOH) and in use for their respective diagnoses at the time, that is, HIT SKK 92 23 and HIT 91 24.

### Intrathecal chemotherapy

Intraventricular therapy was administered to eight patients of cohort A and consisted of either alternating mafosfamide (15 mg) 25 and methotrexate (2 mg/day for 4 days) alternating methotrexate, liposomal cytarabine (25 mg for children ≤3 years, 35 mg for children >3 years) 26 and etoposide 0.25 mg (patients <1 year of age) − 0.5 mg daily for 5 days or alternating liposomal cytarabine and etoposide and varied over time because mafosfamide was no longer available (Fig. 1A and B). Patients treated according to HIT SKK 92 received intraventricular methotrexate only.

### Radiotherapy

Local radiotherapy was applied based on a 3D-conformal treatment plan. Fraction sizes were 1.8 Gy for all target volumes, with a total dose of 54 Gy in most patients. In cohort A, all patients independent of M-stage were treated with local radiotherapy only. The 9-month-old patient (case 4, cohort A) with a high tumor cell count in the cerebrospinal fluid (CSF) and a large spinal metastasis received local irradiation to the posterior fossa and to the lumbar metastasis. In this case, the total dose was limited to 43 Gy to the posterior fossa and 40.6 Gy to the lumbar metastasis.

In cohort B, four patients, 9, 11, 14, and 22 years old at diagnosis, received craniospinal irradiation. Three of five patients treated according to HIT SKK 92 recurred during chemotherapy and none was irradiated (Table 1).

### Toxicity and response evaluation

The Common Terminology Criteria for Adverse Events (version 3.0) was used to grade toxicity of the MUV-ATRT regimen. Neuroimaging studies were performed every 6 weeks to 3 months and CSF was evaluated at each intrathecal chemotherapy. Responses were defined as previously described for standard radiographic criteria 27.

### Statistical analysis

Statistical analyses were conducted according to the procedure of the SPSS 20.0 for Windows^*®*^ package (IBM SPSS Statistics 20.0 für Win.XP/Vista/7, IBM Corporation). Besides descriptive methods, the Kaplan–Meier method was used to estimate the distributions of overall survival (OS) and event-free survival (EFS). Survival was measured from the time of diagnosis to the date of death or last follow-up. In estimating EFS, tumor recurrence, tumor progression, secondary malignancy or death from any cause, whichever developed first, were defined as events. Significance of differences between subgroups was calculated using the log-rank test. A Cox-regression analysis was performed to investigate the impact of the variables—radiotherapy, HDCT, and intrathecal therapy—on OS and EFS. For all tests *P* < 0.05 was considered significant.

### Neuropsychometric assessment of patients treated according to the MUV-ATRT regimen

Neuropsychological data were available for all nine patients treated according to the MUV-ATRT regimen. Four patients were tested before start of chemotherapy and one before HDCT. For four patients, the first full assessment was only possible during follow-up, due to major developmental delay at diagnosis, language problems or very young age. All patients were retested at least once.

The neuropsychological test-battery included age-appropriate Wechsler Scale or a developmental test for patients younger than 3 years of age. In addition, the patients were assessed with age-appropriate tests for attention, memory, processing speed, and visual-spatial perception when possible.

## Results

### Histopathology

The tumors of 22 consecutive patients had characteristic histopathologic and immunohistochemical features and were SMARCB1/INI1 immunonegative. Twelve of these 22 ATRTs were initially diagnosed correctly, whereas 10 were detected retrospectively. Initial diagnoses of the originally misclassified tumors are shown in Table 1.

### Response to treatment

Response was evaluable in a total of 15 patients from both cohorts including patients who had incomplete resection and/or M1–M3 disease. Response is shown in Table 1. Except for patients treated according to the MUV-ATRT regimen patients with residual tumor treated with repeated cycles of PEI (cisplatin 20 mg/m^2^ for 5 days, ifosfamide 1500 mg/m^2^ for 5 days, and etoposide 100 mg/m^2^ for 3 days) chemotherapy achieved the best response suggesting that this combination is particularly effective in ATRTs. However, all three patients treated with PEI chemotherapy and local irradiation did not receive intrathecal therapy and two recurred with leptomeningeal disease without local relapse stressing the importance of intrathecal therapy in focally irradiated patients. In cohort A, all four patients with tumor cells in the CSF treated according to the MUV-ATRT regimen cleared their tumor cells from the CSF and only one patient recurred with leptomeningeal metastases outside of the irradiation field 16 months after diagnosis (Table 1). None of the 16 irradiated patients of both cohorts recurred within the irradiation field.

For the entire cohort of 22 patients the use of radiotherapy was significantly positive for both OS and EFS (*P *<* *0.001 vs. *P *<* *0.001). Similarly, HDCT was associated with a significant positive prognostic value for both OS and EFS (*P *=* *0.018 vs. *P *=* *0.039). Intrathecal chemotherapy had no significant influence on OS and EFS (*P = *0.265 vs. *P *=* *0.917). Cox-regression analyses of these variables showed no significant influence on OS but identified radiotherapy as an independent positive prognostic factor for EFS (*P *=* *0.002).

### Outcome

All nine MUV-ATRT regimen-treated patients are alive for a median of 76 months (range: 16–197), eight in first CR, one patient who developed leptomeningeal metastases outside of the irradiation field 16 months after diagnosis is alive in second CR and off therapy for 12 months. Another patient (case 3) diagnosed with a histopathologically confirmed SMARCB1 nucleopositive glioblastoma within the irradiation field as secondary tumor 9.5 years after diagnosis of his ATRT is also alive.

In cohort B, three patients diagnosed at the age of 35 months, 9, and 22 years, respectively, became long-term survivors, and a fourth patient diagnosed at the age of 11 years has stable disease and is off therapy for 12 months. However, the 9-year-old patient developed a malignant peripheral nerve sheath tumor of the brachial plexus 14.5 years after diagnosis of her basal ganglia ATRT and succumbed to her secondary tumor 16.5 years after diagnosis of her original tumor. The 14-year-old who was treated according to a local protocol in another country died of treatment toxicity.

**Figure 2:**
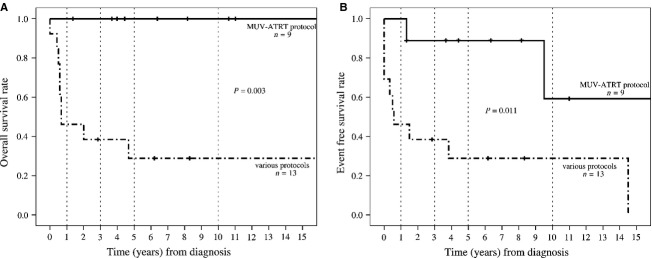
(A) Overall survival (OS) and (B) event-free survival (EFS) of ATRT patients treated according to the MUV-ATRT regimen versus other therapy protocols. OS after 1 year was 100% (MUV) versus 46.2 ± 13.8% (other), after 3 years 100% (MUV) versus 38.5 ± 13.5% (other), and after 5 years 100% (MUV) versus 28.8 ± 13.1% (other). EFS after 1 year was 100% (MUV) versus 46.2 ± 13.8% (other), after 3 years 88.9 ± 10.5% (MUV) versus 38.5 ± 13.5% (other), and after 5 years 88.9 ± 10.5% (MUV) versus 28.8 ± 13.1% (other). MUV-ATRT, Medical University of Vienna ATRT-protocol; ATRT, atypical teratoid rhabdoid tumor.

As of July 2013, 5-year OS for MUV-ATRT-treated patients is 100% and 5-year EFS 88.9 ± 10.5%, and for patients treated with other protocols (cohort B) both, 5-year OS and EFS, are 28.8 ± 13.1% (Fig. 2A and B).

### Feasibility and toxicity of MUV-ATRT chemotherapy

Chemotherapy according to the MUV-ATRT regimen was generally well tolerated and only minor dose adjustments and delays occurred. The most frequent but not unexpected toxicity included bone marrow suppression after cyclophosphamide and vincristine. Great attention was directed toward avoiding febrile neutropenia with consecutive delays in treatment and preemptive IV antibiotics were given during neutropenia. There was one grade 4 mucositis and one grade 3 liver enzyme elevation after methotrexate. Sufficient stem cell harvest was feasible after chemotherapy and cytokine stimulation with G-CSF in all patients. Hematopoietic reconstitution was fast and no graft failure occurred. Following HDCT there was one grade 4 and one grade 3 mucositis, one grade 4 and one grade 3 dermatitis, and one grade 3 liver enzyme elevation. Two patients with cerebellar pontine angle tumors were deaf on one ear (cases 4 and 5) at the time of diagnosis. Five patients developed grade 3 hearing loss (<4 kHz >20 dB) according to the Muenster classification 28 requiring hearing aids, and one patient (case 4) a grade 4 hearing loss (<4 kHz of at least 80 dB) requiring a cochlea implant.

Focal radiotherapy was started no earlier than 6 weeks after HDCT. No problems such as radiation recall, brain edema or necrosis occurred.

### Neuropsychological test results of MUV-ATRT-treated patients

Full IQ scale or total developmental score in nine patients treated according to the MUV-ATRT regimen showed mixed results with four patients scoring within their age norm (mean ± 1 SD; i.e. between 16th and 84th percentile) at all evaluation times (cases 1, 5, 6, and 7). One patient (case 4) improved over time, finally showing age-appropriate results. Four patients scored below age norm (cases 2, 3, 8, and 9) at all evaluation times including one patient (case 2) originally admitted to the pediatric neurology division for evaluation of psychomotor retardation, and two patients (cases 8 and 9) had already attended schools with special educational service prior to diagnosis. Nevertheless, cases 2 and 3 are currently able to attend regular elementary school with special educational service.

## Discussion

While substantial progress has been made in understanding the biology of ATRT, the optimal chemotherapy regimen has yet to be determined, and data supporting a particular combination of agents are lacking. Despite often impressive responses to chemotherapy, the majority of patients in all published studies developed progressive disease early, within 24 weeks of diagnosis, suggesting a rapid development of resistance in ATRTs 15,16,18. The 5-year OS of 56.3 ± 11.3% and EFS of 52.9 ± 11.0% for the whole cohort of 22 consecutive patients treated at our institution compare favorably to all previously published data and seem to have been achieved by the introduction of a prospective uniform treatment protocol (MUV-ATRT regimen), which attained a 5-year OS of 100% and 5-year EFS of 88.9 ± 10.58% in nine patients with an initial correct diagnosis of ATRT.

The role of age at diagnosis has previously been reported important in predicting prognosis 10,12,15. Although the median age of patients treated according to the MUV-ATRT regimen of 24 months was slightly higher than in some other reports, all five patients ≤24 months became long-term survivors and remained in CR from their disease. In most reports, long-term survival in the younger patient cohort was only achieved with early radiotherapy, stressing the importance for this modality also in the younger age group 13,29,30. However, this potentially life-saving treatment option comes at the cost of serious long-term sequelae such as cognitive, motor, visual, and hearing impairment 23,31–33.

In contrast to the published data highlighting the importance of early upfront radiotherapy 13,18,29, our experience with the MUV-ATRT regimen shows that local radiotherapy may be safely postponed for 9 months and deferred to the end of therapy between weeks 38 and 43. All nine patients achieved and/or maintained CR throughout their whole treatment and only one patient recurred with metastases outside of the irradiation field 16 months after diagnosis. It is notable that three patients had M1 stage disease at diagnosis and one 9-month-old had massive tumor cells in the CSF and a spinal metastasis. All four patients with M1–M3 stage tumors were treated with local irradiation only, albeit the latter to both sites with residues suggesting that the combination of intrathecal therapy and systemic HD-MTX, which shows good penetrance into the CSF, is efficacious in eradicating ATRT tumor cells in the CSF. No radiation recall or transverse myelitis as described for three patients treated with a protocol based on a regimen for children with rhabdomyosarcoma with parameningeal extension 18 was observed in any of our patients.

As to the sequence and combination of drugs in our MUV-ATRT regimen, doxorubicin, which was introduced into the multiagent combination based on prior case reports suggesting efficacy 17, was started at a median of 11 days postoperatively (range: 5–28 days). As doxorubicin monotherapy at the proposed dose is not myelosuppressive it does not preclude additional surgery for third ventriculostomy, Ommaya reservoir or shunt placement. The second cycle consisting of cyclophosphamide and vincristine, while myelosuppressive, is usually well tolerated if infection is prevented. Regarding PEI or ICE-type chemotherapy several previous reports suggested that a combination of cisplatin, ifosfamide, and etoposide is efficacious in patients with ATRTs 11,34. This is in accordance with our own observation. Three patients of cohort B receiving PEI chemotherapy courses had a dramatic response to this combination. The only long-term survivor of cohort B diagnosed under the age of 3 years had a partially resected thalamic tumor and achieved complete remission with eight cycles of PEI chemotherapy and focal irradiation. Another patient (case 7 of cohort B) with a spinal ATRT, who had biopsy only and was treated with four cycles of PEI followed by HDCT and irradiation, had a complete response to PEI therapy. He did not receive intrathecal therapy and recurred with metastases without local relapse 18 months after diagnosis, and parents elected to forgo relapse therapy. In retrospect, except for thiotepa during HDCT none of the drugs administered systemically had the potential to penetrate into the CSF and reach cytocidal levels necessary to kill tumor cells floating in the CSF. Similarly, case 8 of cohort B with spinal ATRT, admitted paraplegic and misdiagnosed as Ewing sarcoma, showed a very good partial response by MRI to PEI chemotherapy, and recovered from all neurologic deficits before developing progressive leptomeningeal disease during local irradiation.

HD MTX also used in “Head Start II” 35 appears to be efficacious in ATRT and was well tolerated when given twice in a weekly interval by all young children, and no leukoencephalopathy as evidenced by MRI was observed.

Intrathecal chemotherapy administered via an Ommaya reservoir was incorporated as a method of preventing or treating leptomeningeal dissemination, and appears to be beneficial for patients who receive focal irradiation only. A meta-analysis by Athale et al. 19 showed that intrathecal therapy also made a significant difference in OS (10.5 months vs. 6.5 months, *P *=* *0.011). Intrathecal therapy in the MUV-ATRT-treated patients consisted originally of alternating courses of methotrexate and mafosfamide. When mafosfamide was no longer available, treatment was switched to alternating courses of methotrexate, etoposide, and liposomal cytarabine. Given the good penetrance of systemic methotrexate into the CSF and the potentially increased risk for leukoencephalopathy with additional intrathecal methotrexate, intrathecal methotrexate was omitted in the last three patients of the series.

The role of HDCT in ATRT remains unclear and no definitive conclusions can be made from published data. However, for patients who do not receive radiotherapy HDCT may prevent or delay recurrence as indicated by the results of the “Head Start II” 35 protocol. Furthermore, Finkelstein-Shechter et al. 32 reported on six patients with ATRT who received induction therapy followed by sequential HDCT with autologous stem cell rescue. At a median follow-up of 52 months, four patients were alive without evidence of tumor and three of these patients, including two with metastatic disease were not irradiated. Nicolaides et al. 36 reported on a series of nine consecutive patients with ATRT treated with HDCT and autologous bone marrow transplant and one of the two long-term survivors (98 months from diagnosis) was not irradiated. Similarly, six out of 11 long-term survivors of a Canadian national retrospective study were treated with HDCT without irradiation (median follow-up 38.1 months) suggesting that some patients with ATRT may be spared from radiation 15. In our series, case 9 of cohort B, originally misdiagnosed as CNS PNET, received HDCT as consolidation immediately after HIT SKK 92, albeit no irradiation. She recurred with a combined local and leptomeningeal relapse 46 months after diagnosis.

In conclusion, the OS and EFS rates of our patients provide the best results achieved in patients with ATRTs so far. An initial correct diagnosis, quick start of therapy, adherence to the sequence and dosing of the regimen as well as strict avoidance of delays in therapy appear to be efficacious in preventing early relapses also in young children with M1–M3 stage disease allowing postponement of radiotherapy until after HDCT. Further studies are warranted to confirm the results in a larger cohort of patients and evaluate whether local radiotherapy may be omitted in selected cases.
